# Grateful Workers, Satisfied Workers? A Portuguese Study about Organizational Happiness during COVID-19 Quarantine

**DOI:** 10.3390/bs13020081

**Published:** 2023-01-18

**Authors:** Inês Ataíde, Patrícia Araújo, Alexandra M. Araújo, Rosina Fernandes, Emília Martins, Francisco Mendes

**Affiliations:** 1 Portuguese Institute of Administration and Marketing of Porto, 4450-238 Matosinhos, Portugal; 2 TRIE Center-Transdisciplinary Research Center of Innovation & Entrepreneurship Ecosystems, ISMAT—Instituto Superior Manuel Teixeira Gomes, 8500-656 Portimão, Portugal; 3 Department of Psychology and Education, Portucalense University, 4200-072 Porto, Portugal; 4 Polytechnic Institute of Viseu, School of Education, CI&DEI, 3504-501 Viseu, Portugal

**Keywords:** gratitude, job satisfaction, organizational happiness, internal marketing, human resources marketing

## Abstract

Although work satisfaction has been largely studied, gratitude is an emerging field within multiple sciences, including positive psychology, organizational behavior, and human resources marketing. This ex post facto study aims to characterize gratitude and understand its relations to job satisfaction in a non-probabilistic sample of 521 Portuguese workers (62.2% women), 30.90% and 69.10% in the public and private sector, respectively, mean ages of M = 43, SD = 12.6. Data were collected using anonymous questionnaires during the COVID-19 lockdown. Statistical analyses were performed in SPSS 26, and include Student’s *t*-test, one-way ANOVA, Pearson’s correlations, and a hierarchical linear regression model. Results confirm that Portuguese workers are grateful and satisfied at work. There were statistically significant differences between groups in sociodemographic (*p* < 0.001 and *p* < 0.05), professional (*p* < 0.01 and *p* < 0.001), and perceived living conditions variables (*p* < 0.05) regarding gratitude. Gratitude, alone, explains 8% of job satisfaction. According to the regression model (32.4%), perceptions of satisfaction initiatives and greater job security are also associated with higher levels of job satisfaction (23.6%). Implementation of gratitude-promoting strategies may increase job satisfaction, especially in the post-pandemic period. The investment in workers’ organizational happiness, after the impacts of COVID-19 on work dynamics, is a differentiating organizations success dimension.

## 1. Introduction

An organization’s intangible assets, such as its people, are its most significant competitive differentials [1]. In today’s increasingly ever-changing and unpredictable labor market, organizational happiness, as a construct encompassing different concepts such as job satisfaction, organizational commitment, work involvement, engagement, prosperity, vigor, flow, intrinsic motivation, or positive affect at work, has gained relevance in research and organizations from all types of fields [2,3,4]. 

Employee turnover has costs to the organization, considering recruitment, hiring, and onboarding costs, as well as productivity losses. Merhar [5] predicts that every time a company replaces a salaried employee, it costs an average of 6 to 9 months of salary. Mitrovska and Eftimov [6] confirm these costs, depending on the job. Accordingly, organizations are committing to ensuring long-lasting relationships with their employees, balancing productivity maximization with creating an organization where people want to work [7]. In fact, promoting an institutional climate of happiness and well-being stimulates feelings of satisfaction which, in term, lead to greater commitment, quality, and creativity. Therefore, it is crucial that employees experience job satisfaction [3,8,9]. Positive emotions tend to lead to more favorable attitudes at work, such as organizational commitment, but also greater job satisfaction and, consequently, better performance [10,11].

While salary and compensation are relevant for employees’ satisfaction and retention, several studies [12,13] also stressed the importance of personal characteristics, such as gratitude, for job satisfaction and well-being [14,15,16,17,18]. Gratitude can be defined as an organizational value that can positively affect both individual and collective dimensions of work [19,20,21,22]. 

Organizations and their employees have been dealing with the changes and challenges imposed by the pandemic over the past couple of years. Regardless of the decisions made by companies during the COVID-19 crisis, the way they are communicating and relating to their employees is of utmost importance. It is this connection that will be recalled in people’s memories as an emotionally positive or negative experience, despite all the adversities of the moment [23,24]. Employer branding, happiness, and job satisfaction are built with every small decision, action, or communication [25].

This paper positions itself within a multidisciplinary view, crossing organizational psychology, internal marketing, and organizational behavior to study human experiences at work. The evolution of marketing has come a long way. Throughout history, organizations have been forced to restructure their perspective of action according to the context. In addition, innovation, intensification, and competitive globalization, as well as growing demand and customers’ increasing awareness and access to various products and services, have urged companies to find new strategies to create value and to differentiate themselves from their competitors [26].

In recent years, competitive advantage emerged at an internal level. Motivated employees are also more satisfied, fulfilled, and, consequently, more productive [27]. The concept of internal marketing (IM) was introduced by Berry et al. [28]. This approach appeared as an answer to the problem of consistently providing high standard services, in which quality is the only real factor of differentiation from the competition. Once the customer is more demanding, attracting, retaining, and motivating employees becomes more challenging and focusing on their satisfaction is of utmost importance. To have satisfied customers, a company must also have satisfied employees [28,29].

Over time, the concept evolved. Grönroos [30] proposed a broader perspective of IM: because solely motivating and satisfying employees is not enough, companies must foster customer orientation and sales-mindedness. The author views IM as a method to keep employees motivated and sales-oriented, aware of the individual needs of every customer. George [31] adds that this mindset is boosted by resorting to marketing strategies internally. By including marketing strategies in its definition, the author brings Grönroos’ [30] point of view closer to Berry’s [32]. In conclusion, offering the employee (the internal customer) what companies want to provide their external customers is one of IM’s cornerstones. Once the organization-internal client reflects on the organization-external client relationship, the company is in a better condition to deal with the internal client adequately [33]. 

Considering the growing competition in the corporate world and the search for excellence in individuals, groups, and institutions, organizations have invested in valuing their employees and giving them conditions for improved performance and satisfaction. The premise that happiness can also be achieved at work has encouraged management and organizational behavior researchers to identify effective strategies for its promotion [34]. Research has focused on organizations as contexts for human development and flourishing, as well as individual strengths and positive affect states in organizations [35], following the line of positive psychology proposed by Seligman and Csikszentmihalyi [36]. Thus, happiness, resilience, optimism, and gratitude, among other traits, arise as the object of scientific study in the business field.

Creating value in the short term is no longer enough. To create sustained value in the long term and maximize the profitability of all the assets available, companies need a strategic plan, leading to the identification of organizational happiness (OH) as pivotal for growth and economic results [4,29]. In fact, devaluing their human capital often makes organizations incur in loss of potential development and productivity [3]. Araújo and Fernandes [37] stressed that organizations might experience loss of talent when employees are dissatisfied with their work. The promotion of employees’ happiness is of extreme importance, since positive emotions tend to act as an antidote to negative emotions, and therefore implementing happiness-oriented programs might lead to a less stressed and more satisfied workforce.

Within the different conceptualizations, it is not entirely clear what determines job satisfaction and which impacts it may have. Job satisfaction emerges as a subjective construct different from person to person. However, it seems viable to assume that it derives from a cognitive component, related to the individual’s judgments about work, and an affective or emotional component, related to the way that work makes them feel [38]. In an increasingly globalized world, where every competitive advantage is crucial for organizational development, companies must stimulate their internal customers’ positive emotions and virtues to ensure a sustainable and competitive course of action in the market [4]. Studies that relate job satisfaction and well-being with personal characteristics have been increasing. One of these characteristics is gratitude [19,20,22]. In this paper, we assume gratitude in the McCullough et al. [18] (p. 112) acceptation “as an affective trait that we call the grateful disposition or disposition toward gratitude (…) as a generalized tendency to recognize and respond with grateful emotion to the roles of other people’s benevolence in the positive experiences and outcomes that one obtains”. Gratitude mediates several organizational outcomes, such as the reduction of burnout, performance, well-being, and satisfaction at work and in life; furthermore, demographic variables have no statistical relationship with gratitude [14,15,16,19,20,22,39]. 

Given the lack of evidence regarding the relevance of gratitude for organizational behavior, this study aims to characterize gratitude and understand how it relates to job satisfaction in the Portuguese organizational context amid the COVID-19 pandemic. Specifically, we aim to: (i) study the psychometric characteristics of an instrument for the assessment of gratitude in Portuguese employees; (ii) compare groups in sociodemographic, professional, and perceived living conditions variables during COVID-19 lockdown, regarding gratitude; (iii) explore the relationships between gratitude, job insecurity, and job satisfaction in the Portuguese internal customer; and (iv) explore how a regression model including gratitude, job insecurity, and other sociodemographic, professional, and contextual COVID-related variables, explains which variables influence participants’ job satisfaction. We hypothesized that: (i) there are statistical differences between groups when compared by sociodemographic (sex, marital status, educational levels), professional (sector of activity, employing organization, occupational status), and contextual COVID-related variables (perceived changes in anxiety, stress levels, sleep quality, exercising, meditation); (ii) gratitude has a potential explanation power of the participants’ job satisfaction variance, alone and in a regression model that includes the remaining variables. The conclusions will allow equating intervention strategies to create work contexts that promote job satisfaction.

## 2. Materials and Methods

This is a non-experimental quantitative study with a non-probabilistic convenience sample. Participants were 521 Portuguese employees (62.2% women), with ages ranging from 19 to 68 years (M = 43, SD = 12.6). Most participants (92.5%) had a high-school degree or above (basic education = 7.5%, secondary education = 25.7%, and higher education = 66.8%). Participants worked in their current organization from less than a year to 46 years (M = 17, SD = 11.6). Regarding marital status, 46.8% were single, divorced, separated, or widowed, while the remaining (53.2%) were married or in a civil partnership. Participants self-classified as being low-income (19.19%), middle-class (67.37%), and upper-class (13.44%). Regarding their organization, 30.90% and 69.10% worked in the public and private sector, respectively. The participants’ organizations operated in the primary (1.9%), secondary (10.17%), and tertiary (87.9%) sectors of the economy. A small number of participants were self-employed (6.72%); the remaining participants worked at micro (17.9%), small (19.6%), medium (20.5%), and large (30.7%) organizations.

Participants, selected by snowball sampling techniques, were invited to voluntarily answer the instruments online (via social media), during April and May 2020. Data were collected anonymously, subject to informed consent, included in instrument battery. The administration was pretested to improve data quality and reduce error response. Demographic and occupational information was collected with a questionnaire developed for the purpose of this study. Gratitude was assessed with Gratitude Questionnaire 6 (GQ-6), a six-item scale developed by McCullough et al. [18]. Respondents scored their agreement with each item on a five-point Likert scale, ranging from 1 = “strongly disagree” to 5 = “strongly agree”. The Portuguese translation and validation by Neto [40] also showed adequate reliability of the scale (Cronbach’s *α* = 0.75), similar to our results (Cronbach’s α = 0.76). Job insecurity was evaluated using the Job Insecurity Scale (JIS), a scale of four items developed initially by De Witte [41], and the respondents were asked to rate the items on a five-point Likert scale, ranging from 1 = “strongly disagree” to 5 = “strongly agree”. The Portuguese translation and validation by Chambel and Fontinha [42] reported satisfactory reliability of the scale (Cronbach’s *α* = 0.87). The scale also showed high reliability in the current sample (Cronbach’s α = 0.93). Job satisfaction was measured with the Generic Job Satisfaction Scale, a scale of ten items developed by Macdonald and MacIntyre [43]. Respondents indicated their agreement with each item using a five-point Likert scale, ranging from 1 = “strongly disagree” to 5 = “strongly agree”. The Portuguese translation and validation by Lopes [44] reported a Cronbach’s *α* = 0.79. In the current study, the scale also showed adequate reliability (Cronbach’s α = 0.93).

Data were analyzed with IBM^®^ SPSS^®^ Statistics, version 26.0. Descriptive analyses were conducted to the sample and variables characterization. Exploratory factor analyses and internal consistency were conducted to evaluate the validity and reliability of the instruments. Parametric tests were conducted to compare means between groups, as well as correlations, to study the relations between sociodemographic and occupational variables and the participants’ levels of gratitude. Finally, a hierarchical linear regression model was built to explore how gratitude influenced participants’ job satisfaction.

## 3. Results

### 3.1. The Assessment of Gratitude in Portuguese Employees: Exploratory Factor Analysis and Reliability Analysis of the Gratitude Questionnaire (GQ-6)

The mean of gratitude was 4.03 (SD = 0.68), indicating high gratitude levels in this sample. Job satisfaction presented lower average levels (M = 3.75, SD = 0.70). These results are presented in Table 1.

Exploratory factor analysis of the six items of the GQ-6 was conducted through principal component analysis, with varimax rotation, forcing the extraction of a single factor, as theoretically expected. Considering the Kaiser–Mayer–Olkin (KMO = 0.78) and Bartlett’s Sphericity tests [*χ*^2^ (15) = 1051.37, *p* < 0.001], the data are adequate for factor analysis, with communalities above 0.20 on all items (ranging from 0.21 to 0.74). The extracted factor (Table 2), with eigenvalue = 2.98, explained 49.71% of the total variance. Item loadings on the gratitude factor ranged between 0.46 (item 6) and 0.86 (item 2).

Participants’ answers to the six items of the scale showed an adequate level of internal consistency, with Cronbach’s α = 0.76. The corrected correlations of the items with the total ranged between 0.37 (item 6) and 0.67 (item 2), reinforcing the scale’s reliability. Based on the satisfactory results from validity and reliability analysis, we were able to compute an average score for participants’ gratitude. Levels of gratitude were, on average, high in the current sample (M = 4.03, SD = 0.68), ranging from 2.33 to 5. The asymmetry of −0.30 and the kurtosis of −0.92 suggest that there are no violations of normality in the distribution of responses. In general, we infer that the Portuguese internal customer is grateful.

### 3.2. Relations between Demographic, Occupational and Covid-Related Variables and Gratitude

To explore how gratitude differs between groups in the Portuguese internal client, several Student’s *t*-tests for independent samples and one-way ANOVA analyses with Tukey’s post hoc tests were performed (Table 3). 

Statistically significant differences were found in levels of gratitude between men and women [*t*(519) = −5.78, *p* < 0.001], with women (M = 4.16, SD = 0.65) being more grateful than men (M = 3.82, SD = 0.68). 

Regarding marital status, there are also differences in gratitude levels [*t*(519) = −2.03, *p* < 0.05]. Respondents who were married or in a civil partnership have statistically higher levels of gratitude (M = 4.09, SD = 0.71) than those who are single, widowed, divorced, or separated (M = 3.97, SD = 0.65).

Gratitude also differed based on education levels [*F*(2,518) = 9.41, *p* < 0.001]. Tukey’s post hoc tests showed statistically higher gratitude levels on the respondents who completed higher education (M = 4.12, SD = 0.64) than those who only completed secondary school (M = 3.89, SD = 0.71), *p* = 0.002 and basic education (M = 3.75, SD = 0.78), *p* = 0.004. There are also differences regarding income [*F*(2,518) = 4.58, *p* < 0.05]. The highest income class expresses higher levels of gratitude (M = 4.24, SD = 0.55) compared to the middle class (M = 4.02, SD = 0.68), *p* = 0.032, and to participants with lower incomes (M = 3.93, SD = 0.74), *p* = 0.009.

Occupational parameters also influence levels of gratitude, namely the participants’ sector of activity [*F*(2,511) = 5.65, *p* < 0.01], as well as the dimension of the employing organization [*F*(4,492) = 5.56, *p* < 0.01]. Thus, civil servants (M = 4.18, SD = 0.62) show higher levels of gratitude than those in the private sector (M = 3.96, SD = 0.70), *p* = 0.002, and in large companies (M = 4.23, SD = 0.59) the internal customer also tends to be more grateful than in middle (M = 3.94, SD = 0.73), *p* = 0.008, and small companies (M = 3.87, SD = 0.72), *p* < 0.001. Regarding the economy sector (primary, secondary, and tertiary) [*F*(2,528) = 1.98, *p* = 0.14] and participants’ occupational status (employee, self-employed, working student, and unemployed), there are no statistically significant effects of being part of one of these groups on gratitude [*F*(2,528) = 0.55, *p* = 0.65].

Finally, experiences related to COVID-19 lockdown and social distancing were explored concerning their relations with gratitude. Considering the COVID-19 lockdown, differences in eating habits were significantly related to gratitude [*F*(2,518) = 2.99, *p* < 0.05]: participants who reported to adopt healthier eating habits than before presented higher gratitude levels (M = 4.13, SD = 0.66) than those who reported no changes in their eating habits during the lockdown period (M = 3.94, SD = 0.69), *p* = 0.049, but no differences were found with participants who reported worse eating habits. The same was true for changes in sedentarism [*F*(2,518) = 3.18, *p* < 0.05]: participants who reported sitting time decreased during the lockdown presented statistically higher levels of gratitude (M = 4.12, SD = 0.69) than those who perceived no changes in their sedentarism (M = 3.90, SD = 0.72), *p* = 0.032. There were no differences regarding comparisons with participants who reported worse sedentarism habits. Perceived changes in anxiety and daily stress were also related to gratitude [*F*(2,518) = 6.91, *p* < 0.01]: participants who reported their anxiety and stress levels decreased during the lockdown presented higher levels of gratitude (M = 4.17, SD = 0.67) than those who reported no perceived changes (M = 3.87, SD = 0.69), *p* = 0.001. In turn, there were no statistically significant differences in gratitude levels based on sleep quality [*F*(2,518) = 2.4, *p* = 0.09], exercising [*F*(2,518) = 0.72, *p* = 0.49], and meditation or similar activities [*F*(2,518) = 0.09, *p* = 0.91].

### 3.3. Relations between Gratitude, Job Insecurity, and Job Satisfaction during the COVID-19 Lockdown Period

Perceptions of job insecurity collected during the COVID-19 lockdown correlated negatively with gratitude (*r* = −0.12, *p* < 0.01) and job satisfaction (*r* = −0.25, *p* < 0.001). Therefore, workers who felt more secure reported higher levels of gratitude and job satisfaction. In addition, gratitude correlated positively with job satisfaction (*r* = 0.28, *p* < 0.001): workers who reported higher levels of gratitude also reported higher levels of job satisfaction. Correlation results are presented in Table 4. 

In a hierarchical multiple regression (Table 5), potential predictors of job satisfaction were entered in blocks. Gender, marital status, education level, income, and the organization’s activity sector and dimension were entered first but showed no significant predictive power. The organizations’ investment in satisfaction promotion programs (yes or no) and job insecurity were entered in the second block, showing they are both significant predictors of job satisfaction (*β* = 0.43, *p* < 0.001; *β* = −0.18, *p* < 0.001, respectively), contributing together to the explanation of 23.6% of the job satisfaction variance. Finally, gratitude was entered in the third block and was identified as a positive predictor of job satisfaction (*β* = 0.304, *p* < 0.001), explaining, by itself, 8.3% of the variance of job satisfaction. Altogether, the three blocks allow the explanation of 32.4% of the total variance of job satisfaction. In sum, the organizations’ initiatives for satisfaction promotion, more positive perceptions of job security, and higher levels of gratitude were associated with higher levels of job satisfaction in our sample. 

## 4. Discussion

The present investigation explored the levels of gratitude in a non-probabilistic sample. Thus, although sample limitations require prudence in the generalization possibilities, we concluded that gratitude and happiness are found together in Portuguese workers, despite data collection during the pandemic crisis. Also, there is a positive relationship between these two dimensions in these Portuguese participants.

Concerning sociodemographic data, higher levels of gratitude were declared by women, married participants, and participants with higher levels of education and income. Regarding people with and without children and those living alone or with others, there were no statistically significant differences. Neto [40] found no relationship between sociodemographic factors and gratitude. Regarding professional variables, the sector of activity and the size of the organization were shown to influence the levels of gratitude. Public employees showed higher levels of gratitude than private employees and those working in large companies were more grateful than those who work for small and medium-sized companies. Living conditions allow us to conclude for lower levels of gratitude in people who lived alone during lockdown. On the contrary, declared gratitude was higher for those who, during this period, opted for a healthier diet and for those who saw their sitting time and their daily anxiety and stress decrease. 

Finally, gratitude values are above the midpoint of the scale and, therefore, the Portuguese internal client is considered grateful. Despite the data collection in times of crisis, isolation, and uncertainty, when compared to previous studies, the Portuguese are among the most grateful [21,45,46], only surpassed by the sample of Italian civil servants by Cortini et al. [47].

According to the evidence, gratitude, as an emotion that can be stimulated by intervention or as a personality trait, tends to increase the satisfaction felt at work. Thus, it was also found that the internal customer has a positive job satisfaction. Despite lower job satisfaction than gratitude, job satisfaction values are above the midpoint of the scale. Despite being slightly less satisfied compared with the sample by Cortini et al. [47], values of job satisfaction obtained were clearly superior to those of studies in the East [45,46]. Stegen and Wankier [21] had the same value for job satisfaction in Americans prior to the gratitude-inducing intervention they carried out in their study.

We found a moderate correlation [48] of 0.28 between gratitude and job satisfaction, which is less strong than those exposed by Kim et al. [45], but greater than the findings of Cortini et al. [47], who found no statistical relationship. The findings showed the lowest score for the salary, interconnecting with Cardoso’s [49] dimension of internal marketing, but different from the focus of this research (organizational happiness).

After controlling for the effects of variables that had previously been found to significantly influence levels of gratitude, the organizations’ investment in programs/initiatives that promote satisfaction and job insecurity proved to be predictors of job satisfaction. Gratitude alone is a moderately significant predictor of job satisfaction, explaining 8.3% of the total variance. These results, while similar to those reported in literature [35] about the influence of positive personal characteristics on different organizational outcomes, seem even more solid. In conclusion, perceptions of organizational investment in initiatives that promote satisfaction, greater job security, and higher levels of gratitude are associated with higher levels of job satisfaction.

We know that salary and compensation represent only a small part of job satisfaction [13]. Fair payment and the dignification of the human being inorganizations’ processes are just the beginning of promoting an overall positive organizational culture, recognition, and wellbeing, with the profession of CHO’s—Chief Happiness Officers on the rise and the search for positive meaningful work becoming one of the next human challenges for the future [37].

Regarding the analysis of organizational practices that best promote the development of gratitude levels, one suggestion would be to first identify the activities to be analyzed and collect independent samples of each one. This would allow a detailed and isolated analysis of the regularity variable and, in a comparison between the various samples, the identification of determinants in an activity that promotes higher levels of gratitude.

However, “wanting happiness 5.0 and living in a society 2.0, with education 2.0 and organizational management 1.0 is clearly a challenge for some communities/countries” [50] (p. 25). Our results seem promising, as they point to the relevance of the merger between areas of study of organizational happiness, namely marketing, psychology, sociology, and even organizational philosophy. Different scientific areas must come together and collaborate in the proposal of diagnostic and intervention strategies that promote happy human beings, also in the work context. We highlight teambuilding strategies (radical activities, tourist, and cultural experiences) to promote cohesion and well-being in workers. 

The findings corroborate the literature as showed in a recent systematic review study [51], nevertheless it remains important to explore mediating variables in the gratitude and job satisfaction relationship [52].

Finally, as the main limitation, the procedure used in data collection stands out for not allowing control over the conditions of selection of participants and response to the instruments, given the COVID-19 pandemic. It is important to replicate the study in the post-pandemic period, but also with larger samples and improving the data collection process. Further, we highlight the value of objective assessment instruments instead of self-reports on experiences related to COVID-19 lockdown. It would be critical to understand the extent of these results, at the European level, as well as worldwide, to assess the effects of levels of development on the studied variables.

## Figures and Tables

**Table 1:** Descriptive statistics of the Gratitude Questionnaire and Job Satisfaction Scale.

	Min.	Max.	M	SD
Gratitude	2.33	5.00	4.03	0.68
Job Satisfaction	1.40	5.00	3.75	0.70

**Table 2:** Principal Components Analysis of the Gratitude Questionnaire (QQ-6; N = 521).

Item	Factor Loading	*h^2^*
1	0.84	0.70
2	0.86	0.74
3	0.53	0.28
4	0.73	0.53
5	0.72	0.52
6	0.46	0.21
Eigenvalue	2.98	
% Explained Variance	49.71	

**Table 3:** Gratitude results according to demographic, occupational, and COVID-related variables.

Independent Variables	M	DP	*t/F*	*p*
Sex			−5.78	<0.001
Women	4.16	0.65		
Men	3.82	0.68		
Marital status			−2.03	<0.05
Single/widowed/divorced	3.97	0.65		
Married/civil partnership	4.09	0.70		
Education levels			9.41	<0.001
Basic education	3.75	0.78		
Secondary school	3.89	0.71		
Higher education	4.12	0.64		
Income			4.58	<0.05
Higher	4.24	0.55		
Middle	4.02	0.68		
Lower	3.93	0.74		
Sector of activity			5.65	<0.01
Government	4.18	0.62		
Private	3.96	0.70		
Organization dimension			5.56	<0.01
Large	4.23	0.59		
Middle	3.94	0.73		
Small	3.87	0.72		
Eating habits during COVID-19			2.99	<0.05
Healthier	4.13	0.66		
No changes	3.94	0.69		
Sedentarism during COVID-19			3.18	<0.05
Decreased sitting time	4.12	0.69		
No changes	3.90	0.72		
Anxiety/daily stress during Covid			6.91	<0.01
Lower	4.17	0.67		
Higher	3.87	0.69		

**Table 4:** Correlations (significative) between gratitude, job satisfaction, and job insecurity.

	Gratitude	Job Satisfaction	Job Insecurity
Gratitude	1	0.28 ***	−0.12 **
Job satisfaction		1	−0.25 ***
Job insecurity			1

** *p* < 0.01; *** *p* < 0.001.

**Table 5:** Hierarchical multiple regression for potential predictors of job satisfaction.

Predictors	β	SD (β)	β	R^2^	∆R^2^
Block 1					
Gender	−0.02	0.06	−0.01		
Marital status	0.00	0.05	0.00	0.01	0.01
Education level	−0.06	0.05	−0.05		
Income	0.00	0.05	0.00		
Block 2					
Activity sector	−0.00	0.05	0.00	0.24	0.24
Dimension	−0.00	0.02	−0.01		
Investment in satisfaction programs	0.17	0.02	0.43 ***		
Job insecurity	−0.09	0.02	−0.18 ***		
Block 3					
Gratitude	0.30	0.04	0.30 ***	0.32	0.08

Note. *** *p* ˂ 0.001.

## Data Availability

Data supporting reported results can be found in the master thesis available, in paper, at the IPAM (Instituto Português de Administração de Marketing), and mentioned in the bibliographic references in the author’s name Inês Ataíde, which results are an integral part.
